# Supplementary material for: The Antidiabetic Agent Acarbose Improves Anti-PD-1 and Rapamycin Efficacy in Preclinical Renal Cancer
Source: Cancers (Basel). 2020 Oct 6;12(10):2872. doi: 10.3390/cancers12102872 (PMC7601245; doi:10.3390/cancers12102872)
Supplement: Supplementary file 1 [file cancers-12-02872-s001.pdf]

cancers

MDPI

Supplementary Materials

The Antidiabetic Agent Acarbose Improves Anti-PD-1 and Rapamycin Efficacy in Preclinical Renal Cancer

Rachael M. Orlandella, William J. Turbitt, Justin T. Gibson, Shannon K. Boi, Peng Li, Daniel L. Smith Jr. and Lyse A. Norian

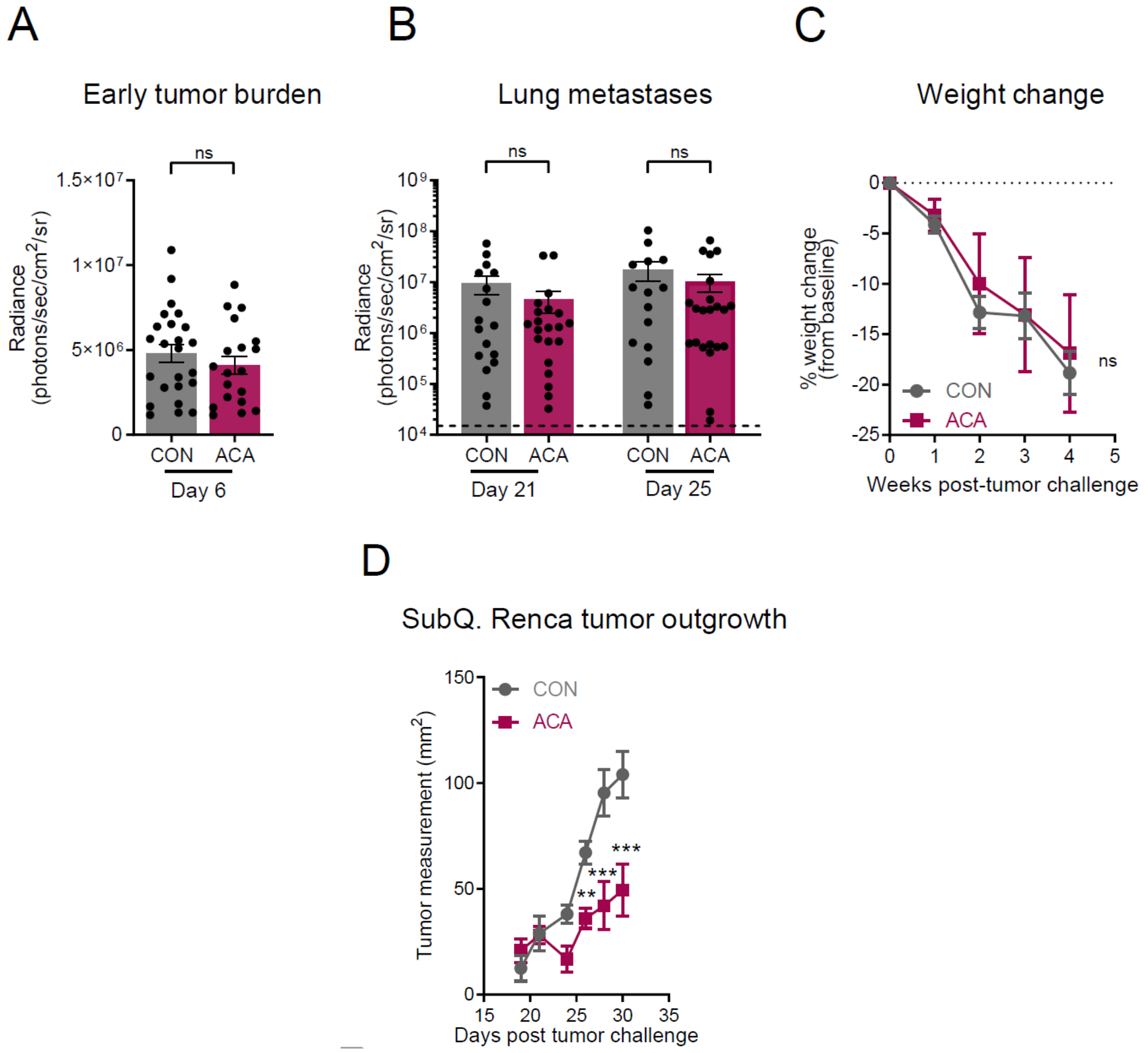

Figure S1. Acarbose does not alter early tumor burden, lung metastases, or weight loss. (**A**) BLI quantification of day 6 primary renal tumor burdens. (**B**) Day 21 BLI quantification of day 21 and 25 excised metastatic lungs. Dotted black line represents the average BLI of tumor-free lungs. (**C**) Percent weight change over time versus baseline body weights. (**D**) Tumor areas measured by caliper over time of Renca tumor cells injected subcutaneously. Data from individual mice from at least 3 experiments are shown in **A** and **B.** Data in **C** and **D** are summary data from 2 experiments with n = 6-24 mice per group. Data are presented as means ± SEM. Statistical differences were determined using nonparametric Mann-Whitney tests in **A** and **B**. Statistical differences in **C** and **D** were determined through two-way repeated measures or mixed models ANOVA (ns = not significant; * = $p < 0.01$; *** = $p < 0.001$). CON = Control diet; ACA = Acarbose-containing diet.

A

Diet	Composition
5LG6 Control diet (5LG6 CON)	20.5% kcal protein<br>12.1% kcal fat<br>67.4% kcal carbohydrates
5LG6 Acarbose-containing diet (5LG6+ACA)	20.5% kcal protein<br>12.1% kcal fat<br>67.4% kcal carbohydrates<br>0.1% Acarbose

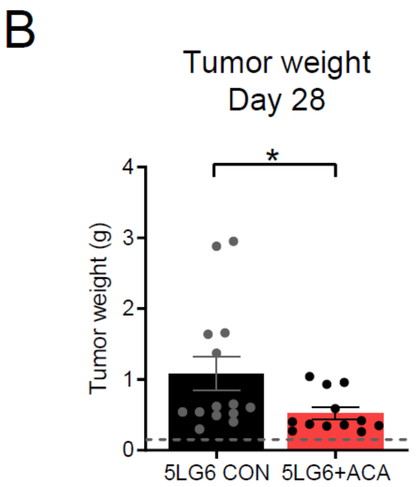

Figure 2. Acarbose impairs renal tumor growth in mice fed the 5LG6 diet composition. (**A**) Macronutrient composition percentages in kilocalories (kcal) of 5LG6 CON and 5LG6 + ACA diets. (**B**) Day 28 excised renal tumor weights. Dotted gray line represents the average weight of tumor-free kidneys.Data in **B** are from 2-3 independent experiments and data from individual mice are shown. Data are presented as means ±SEM. Statistical differences were determined using nonparametric Mann-Whitney tests (* = $p < 0.05$). 5LG6 CON = 5LG6 Control diet; 5LG6 + ACA = 5LG6 Acarbose-containing diet.

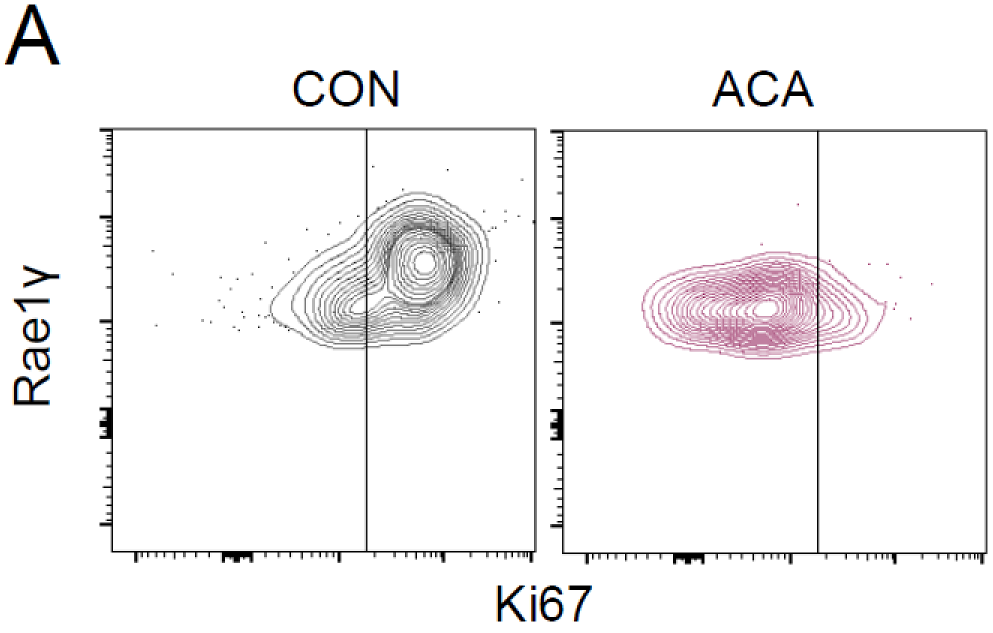

Figure S3. Renal tumor cells from day 21 tumors exhibit less Ki67 staining with acarbose. (**A**) Representative Ki67 flow cytometry plots from day 21 renal tumors (*Ancestry: FSC-A vs SSC-A > Single cells > Live > CD45-Rae1γ+*). CON = Control diet; ACA = Acarbose-containing diet.

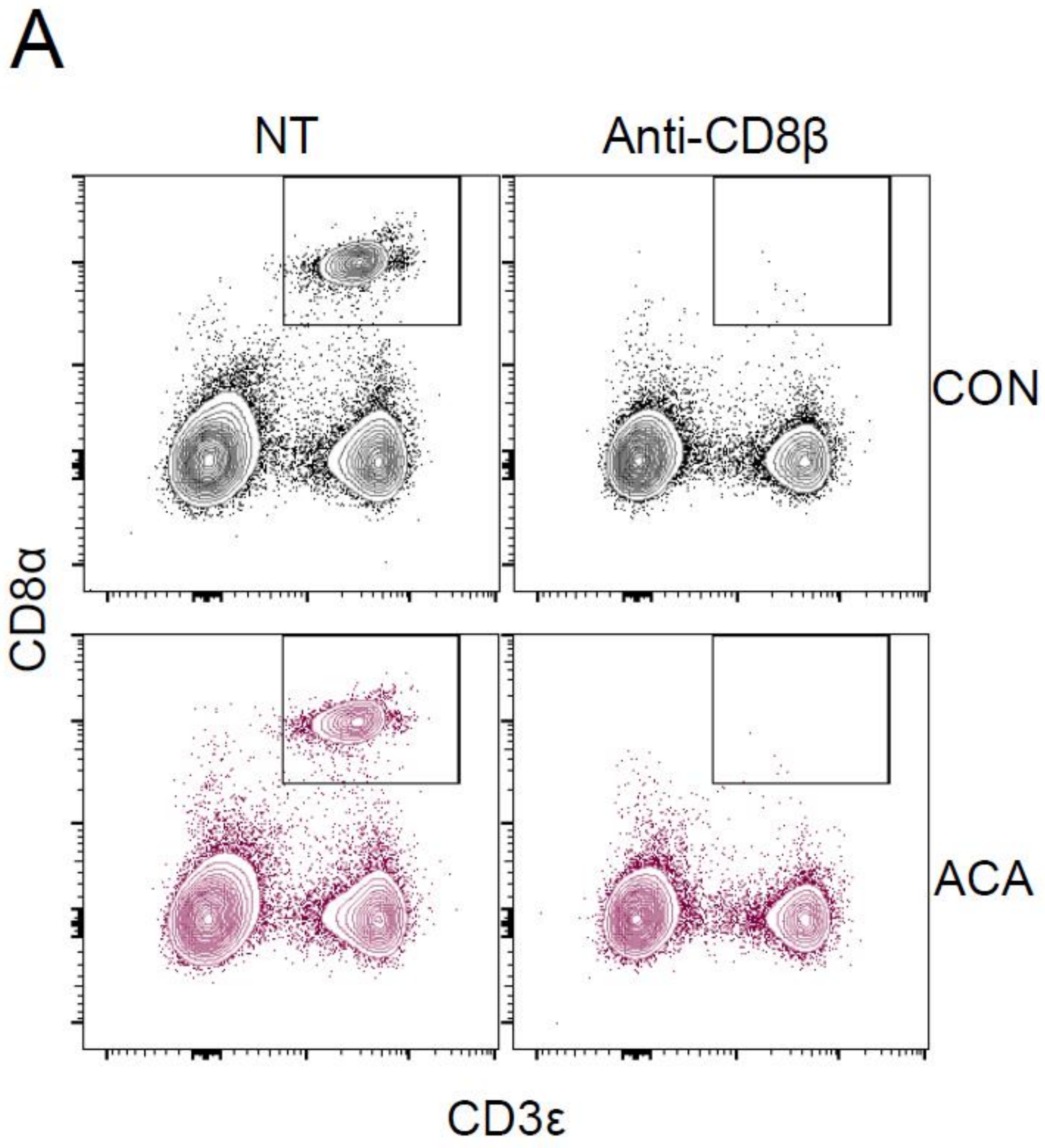

Figure S4. Depletion of CD8 T cells in mice on CON and ACA. (**A**) Representative flow cytometry plots from day 21 spleens from CD8-intact mice and following CD8 depletion with anti-CD8β. (*Ancestry: FSC-A vs SSC-A* > *Single cells* > *Live*). CON = Control diet; ACA= Acarbose-containing diet; NT = no treatment.

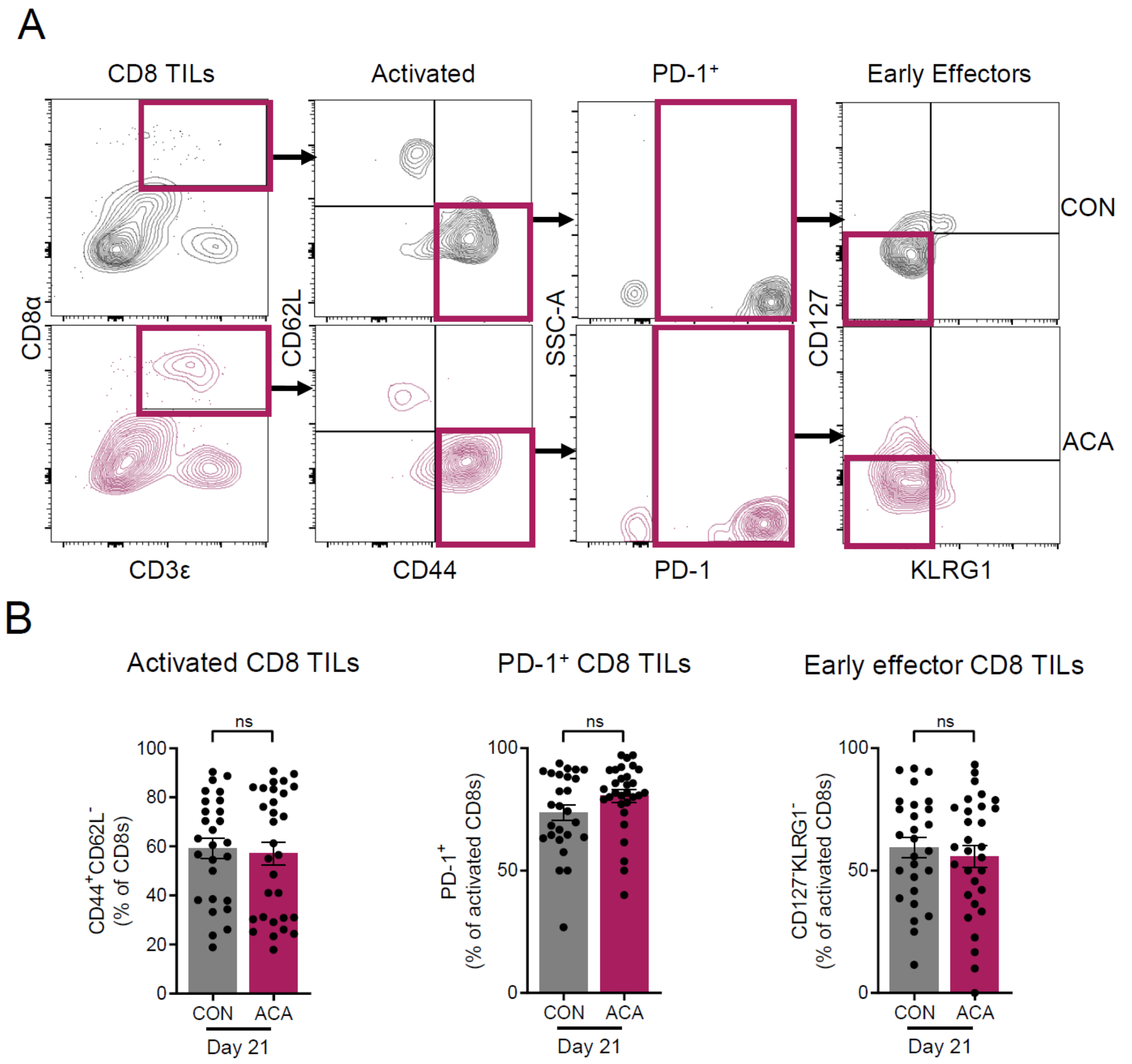

Figure S5. CD8 TILs from mice on CON and ACA exhibit similar phenotypes. (**A**) Representative gating strategies from day 21 renal tumors to define activated ($CD44^{+}CD62L^{-}$), PD-1+, and early effector populations ($CD127^{-}KLRG1^{-}$). (**B**) Pooled data from activated, PD-1+ and early effector populations from day 21 renal tumors. Data from individual mice from at least 3 experiments are shown**.** Data are presented as means ± SEM. Statistical differences were determined using t-tests or nonparametric Mann-Whitney tests. CON = Control diet; ACA= Acarbose-containing diet. ns = not significant.

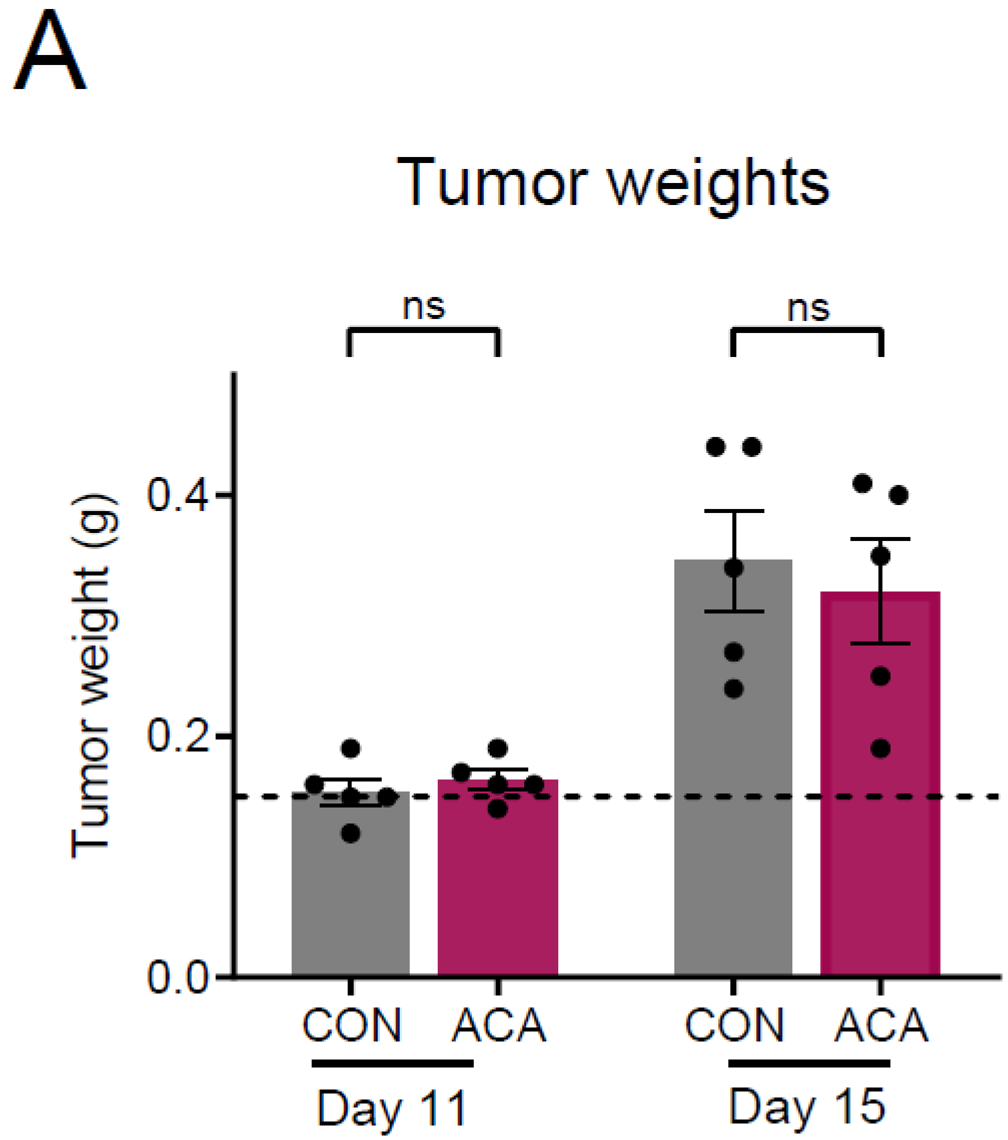

Figure S6. Renal tumor weights are similar with ACA in the early stages of tumor growth. (**A**) Excised renal tumor weights from days 11 and 15. CON = Control diet; ACA = Acarbose-containing diet. Data from individual mice from at least 1 experiment per time point are shown. Data are presented as means ± SEM. Statistical differences were determined using nonparametric Mann-Whitney tests (ns = not significant). CON = Control diet; ACA= Acarbose-containing diet.

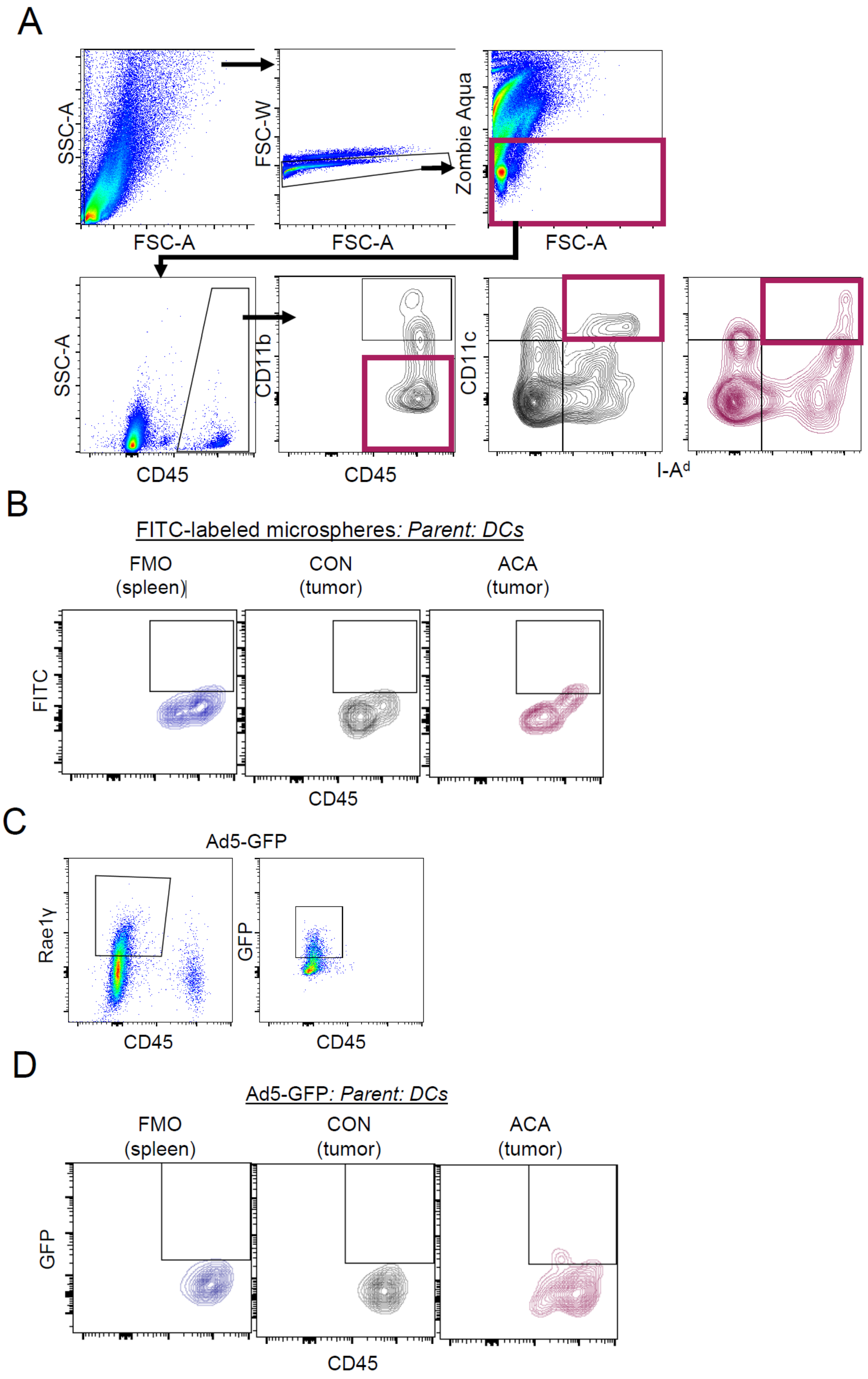

Figure S7. Defining dendritic cell subsets and GFP expression in day 11 renal tumors (**A**) Gating strategy from day 11 renal tumors for defining dendritic cells (DCs). (**B**) Intratumoral DC expression of FITC. (**C**) Day 11 tumor cell expression of GFP. (**D**) Intratumoral DC expression of GFP.

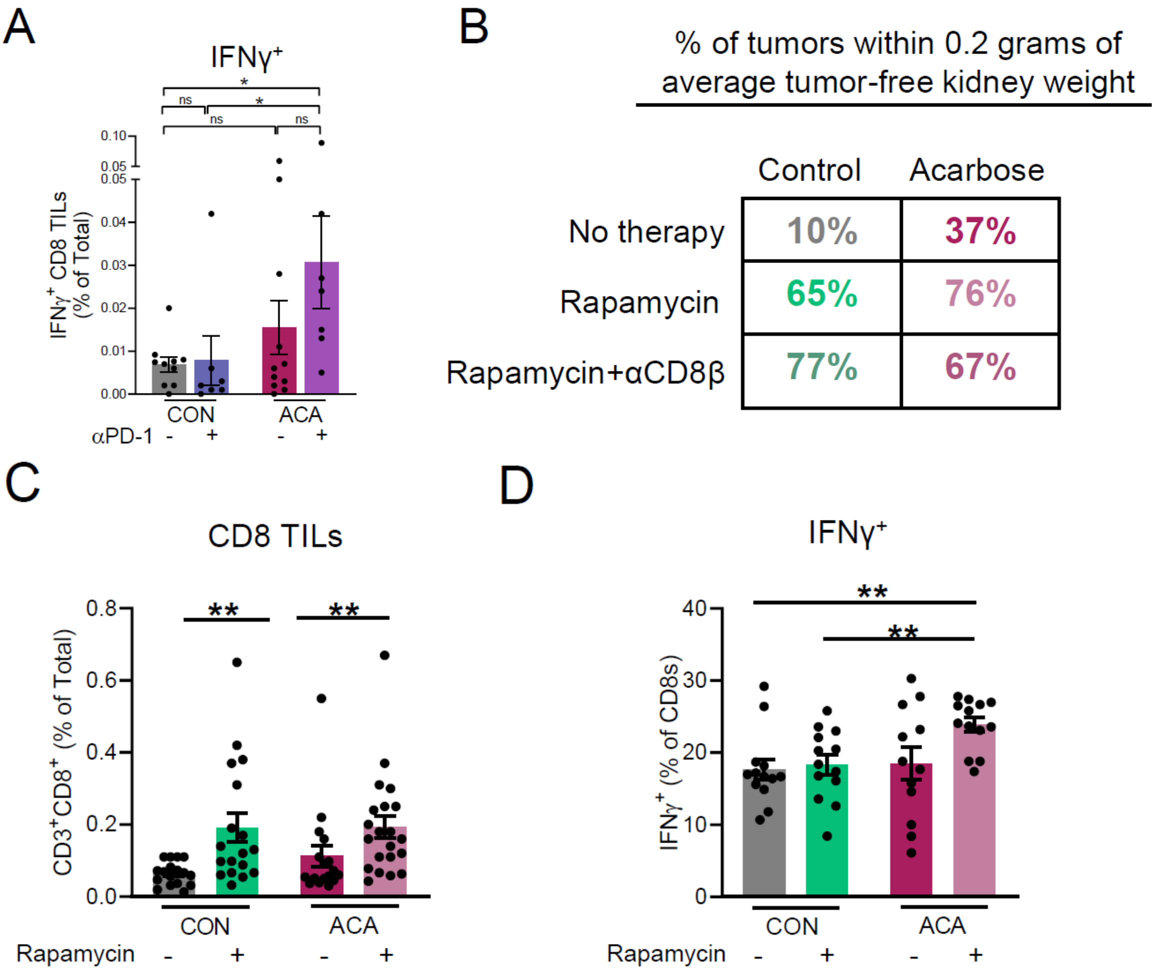

Figure 8. Combinatorial therapeutic outcomes with ACA + anti-PD-1 or rapamycin. (**A**) Frequency of IFNγ+CD8 T cells within day 21 renal tumors as a frequency of total cells evaluated (*Ancestry: FSC-A vs SSC-A > Single cells > Live, CD11b-CD11c-dump gate > CD3*$^+$*CD8*$^+$). (**B**) Percentages of tumors within 0.2 grams of a tumor-free kidney across all groups from rapamycin experiments. (**C**) Frequency of CD8 T cells within day 21 renal tumors as a frequency of total cells evaluated (*Ancestry: FSC-A vs SSC-A > Single cells > Live, CD11b-CD11c-dump gate*) (**D**) Frequencies CD8 TILs producing of IFNγ$^+$ within day 21 renal tumors (*Ancestry: FSC-A vs SSC-A > Single cells > Live, CD11b-CD11c-dump gate > CD3*$^+$*CD8*$^+$*)*. Data from individual mice are shown and are from 2-4 independent experiments. Data are presented as means ± SEM. Statistical differences were determined using one-way ANOVA with Tukey's post hoc test or nonparametric Kruskal-Wallis ANOVA with Dunn's post hoc test. (ns = not significant; *$p < 0.05$, **$p < 0.01$). CON = Control diet; ACA = Acarbose-containing diet.

© 2020 by the authors. Licensee MDPI, Basel, Switzerland. This article is an open access article distributed under the terms and conditions of the Creative Commons Attribution (CC BY) license (http://creativecommons.org/licenses/by/4.0/).
